# Comparative efficacy and safety of systemic therapy for advanced hepatocellular carcinoma: a systematic review and network meta-analysis

**DOI:** 10.3389/fonc.2023.1274754

**Published:** 2023-12-06

**Authors:** Di Wu, Binyang Jia, Muyuan Jia, Haitao Zhao, Hong Zhao, Jinxue Zhou

**Affiliations:** ^1^ Department of Otolaryngology, Head and Neck Surgery, Graduate College of Youjiang Medical University for Nationalities, Baise, Guangxi Zhuang, China; ^2^ Department of Hepatobiliary and Pancreatic Surgery, Affiliated Cancer Hospital of Zhengzhou University, Zhengzhou, Henan, China; ^3^ Department of Neuro-Oncology, Beijing Arion Cancer Center, Beijing, China; ^4^ Department of Liver Surgery, State Key Laboratory of Complex Severe and Rare Diseases, Peking Union Medical College Hospital, Chinese Academy of Medical Sciences and Peking Union Medical College, Beijing, China; ^5^ Department of Hepatobiliary Surgery, National Cancer Centre/National Clinical Research Centre for Cancer, Cancer Hospital, Chinese Academy of Medical Sciences and Peking Union Medical College, Beijing, China

**Keywords:** hepatocellular carcinoma, targeted therapy, tyrosine kinase inhibitors, immune checkpoint inhibitors, first-line treatment, second-line treatment

## Abstract

**Background:**

In recent years, there has been rapid development in systemic therapeutic agents for advanced hepatocellular carcinoma. However, most treatment modalities lack head-to-head comparisons, and the distinctions in their efficacy and safety have yet to be elucidated. Consequently, the accurate selection of a treatment regimen poses a significant challenge for clinicians.

**Methods:**

This study incorporated twenty-three randomized controlled trials, encompassing fifteen first-line and eight second-line treatments, and involving a total of 14,703 patients with advanced hepatocellular carcinoma. Results: In the context of first-line treatment, it was observed that the combination of a PD-1 inhibitor with bevacizumab (1/15) significantly extended overall survival in patients with advanced HCC. Furthermore, PD-1 inhibitors combined with TKIs (1/15) and PD-1 inhibitors combined with bevacizumab (2/15) exhibited enhanced efficacy in reducing the risk of progression-free survival events. In second-line therapy, the network meta-analysis revealed that all investigational agents prolonged progression-free survival in patients with advanced hepatocellular carcinoma when compared to placebo. Cabozantinib ranked first (1/7) in this regard. However, this translated into an overall survival benefit only for cabozantinib, regorafenib, ramucirumab, and pembrolizumab, with regorafenib achieving the highest ranking (1/7).

**Conclusion:**

In the treatment of advanced HCC, the immune checkpoint inhibitor combined with bevacizumab regimen and the immune checkpoint inhibitor combined with TKI regimen stand out as the two most effective first-line treatment options. It is noteworthy that, for patients with absolute contraindications to VEGF inhibitors, dual immunotherapy is the preferred choice. For second-line treatment, regorafenib and cabozantinib are identified as the two most effective options.

**Systematic review registration:**

https://www.crd.york.ac.uk/prospero, identifier CRD42023440173.

## 1 Introduction

Hepatocellular carcinoma (HCC) is the sixth most common malignant tumor and the third most fatal tumor worldwide (1). Due to the insidious onset of HCC, the majority of patients are in advanced stages at the time of initial diagnosis. Surgical resection, radiofrequency ablation, and hepatic artery chemoembolization (TACE) have limited therapeutic effects on patients with advanced HCC, and the 5-year survival rate is less than 20% (2, 3). In recent years, with the deepening of research on tumor molecular signaling pathways and tumor microenvironment, the treatment of advanced HCC has developed rapidly, and a variety of targeted therapies and immunotherapies have been successively applied to the clinic, as well as the combination of the two have been successively approved for the treatment of advanced HCC, which has brought more clinical benefits to the patients (4).

In November 2007, the U.S. FDA approved sorafenib for the first-line treatment of advanced HCC based on the results of the SHARP clinical trial (5), and it remained the only evidence-based treatment for advanced HCC for the next 10 years. In recent years, due to the continuous exploration of the pathogenesis of hepatocellular carcinoma, a large number of phase III randomized controlled clinical trials have flooded into the clinic, for example, the results of the phase III REFLECT clinical trial suggested that compared with sorafenib, lenvatinib reached the non-inferior trial endpoints in terms of overall survival (OS), and the patients’ progression free survival (PFS) and objective response rate (ORR) were significantly improved, therefore, lenvatinib was approved to be used as first-line treatment for intermediate and advanced stage HCC in 2018 (6). And then clinical trials were conducted for donafenib (7), as well as the second-line drugs regorafenib (8), cabozantinib (9), ramucirumab (10, 11) and apatinib (12). In addition, the emergence of atezolizumab in combination with bevacizumab ushered in the era of combined immune checkpoint inhibitors (ICIs) combined with antiangiogenic drug therapy, establishing a new standard of first-line therapy (13). Following IMbrave150, a variety of ICIs have been explored in combination with anti-angiogenic drugs. For example, in the ORIENT-32 phase III clinical trial, anti-programmed death 1 (anti-PD-1) in combination with an anti-VEGF receptor antibody has been shown to prolong median OS, PFS, and improve ORR in advanced HCC (14). Although the COSMIC-312 clinical trial (15) (atezolizumab in combination with cabozantinib) and the LEAP-002 (16) clinical trial (pembrolizumab in combination with lenvatinib) did not meet the expected OS endpoints, they were still partially efficacious and showed some clinical activity. The results of SHR-1210-III-310 showed (17) that the camrelizumab combined with apatinib regimen met its primary efficacy endpoints in patients with advanced HCC, further suggesting that TKIs are the best combination partners for the treatment of ICIs. In addition, results from the HIMALAYA clinical trial showed that anti-programmed death ligand 1 (anti-PD-L1) plus anti-cytotoxic T-cell lymphocyte antigen 4 (anti-CTLA-4) therapy prolonged OS in advanced HCC (18).

Currently, there is a wide variety of treatment options for advanced HCC, their clinical outcomes are mixed, and there is a lack of head-to-head comparisons between most of the treatment options, so how to choose the optimal first- and second-line treatment options is an urgent clinical problem to be solved. Therefore, this network meta-analysis (NMA) aims to compare the efficacy of different therapeutic agents in patients with advanced HCC with different lines of treatment.

## 2 Materials and methods

### 2.1 Search strategy and selection criteria

According to the Preferred Reporting Items for Systematic Reviews and meta analysis (PRISMA) guidelines (19), we searched PubMed, Embase and Cochrane library databases. The search strategy is shown in the **Supplemental Table 1**. To minimize search bias, abstracts from American society of clinical oncology (ASCO) and European society for medical oncology (ESMO) were also searched as a supplement, and the search was conducted until December 1, 2022, with the restriction that the language was English. The inclusion criteria for this study were: patients with advanced (unresectable or metastatic) HCC comparing the efficacy of anti-angiogenic drugs, ICIs, or their combination in HCC, and anti-angiogenic drug therapy including TKI/monoclonal antibodies. The primary study endpoint of this study was OS, and the secondary study endpoints were PFS, ORR, and grade 3 or higher adverse events (≥3AE). Included studies included phase III randomized controlled trials (RCTs) in first and second line. Exclusion criteria were: combination therapy studies other than those mentioned above, including phase I and phase II clinical trials and trials with incomplete data re-porting.

### 2.2 Data analysis

Statistical analysis was performed using R statistical software (version 4.1.3). For OS, PFS survival endpoints, risk ratios (HR) and 95% confidence intervals (CI) were extracted from the included clinical trials, which were log-transformed for the presentation of results. For ORR, AE, the estimated odds ratios (OR) was calculated to compare treatments. Fixed-effects model or random-effects model was selected based on the level of heterogeneity of the study data, which was quantified using the I² statistic; if I² > 50%, indicating significant heterogeneity of the data, the random-effects model was used, otherwise the fixed-effects model was used. Each outcome metric was analyzed to obtain the surface under the cumulative ranking (SUCRA), which is used to rank interventions, with larger values suggesting better outcomes when assessing medication effectiveness (20). The opposite result was observed when assessing the safety (20, 21). In addition, mapping the web of relationships for each of the outcome indicators. When a closed loop exists, in-consistency detection is performed using the point score method (22) with a test level of α= 0.05. Risk of bias in trials was assessed using the tool recommended by the Cochrane Handbook 6.0 (23), which evaluates aspects in the following main areas: randomized sequence generation, allocation concealment, blinding, incomplete outcome data, and selective outcome reporting. Two reviewers independently assessed the quality of the study trials and resolved disagreements by recommending a third reviewer.

## 3 Results

**Figure 1:**
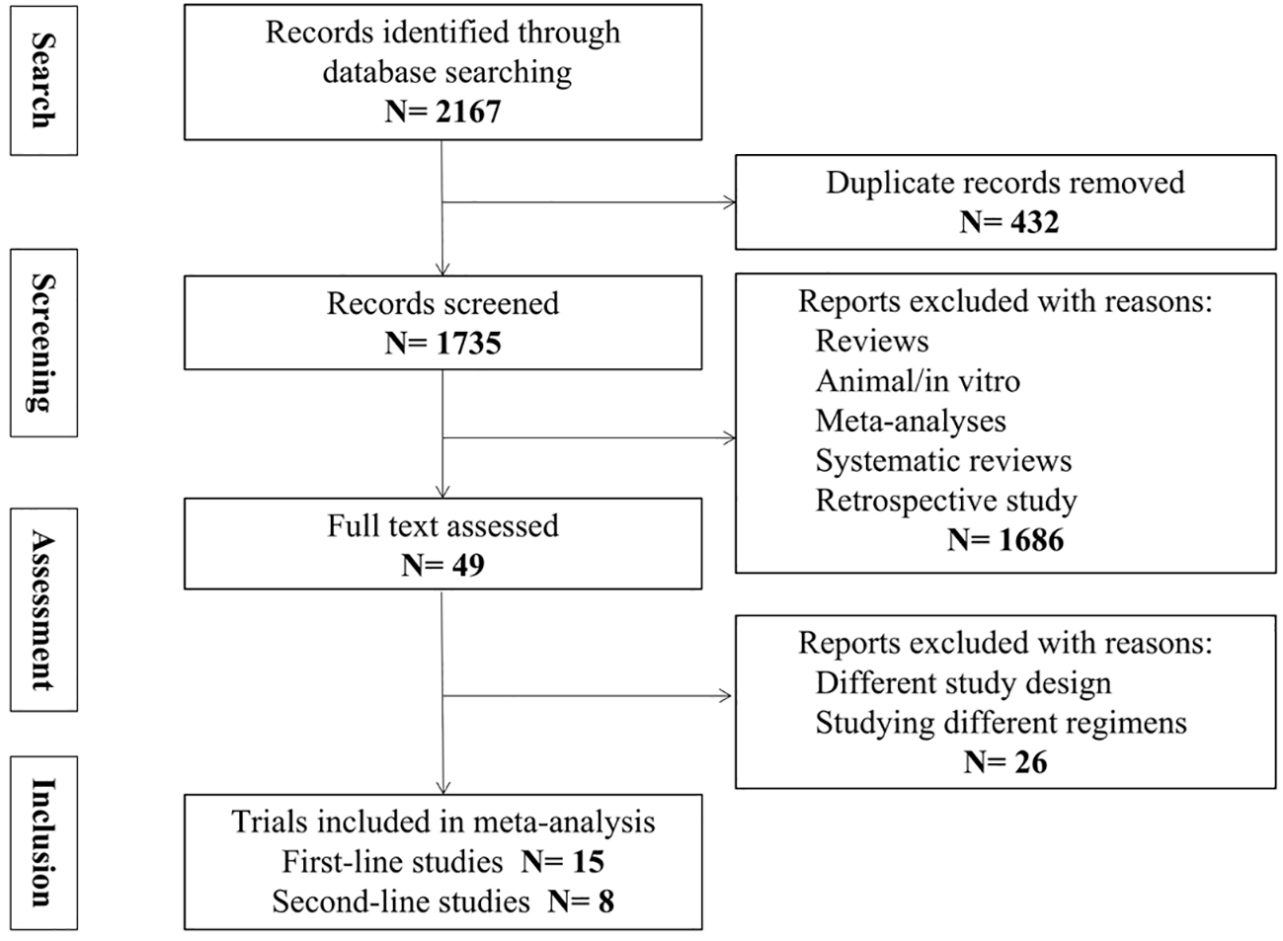
PRISMA flow diagram showing screening and selection process.

The initial search for this study yielded 2,167 articles, and 23 articles were finally included, of which 15 were first-line studies and 8 were second-line studies (**Figure 1**).

### 3.1 First-line treatments

#### 3.1.1 Baseline characteristics

**Figure 2:**
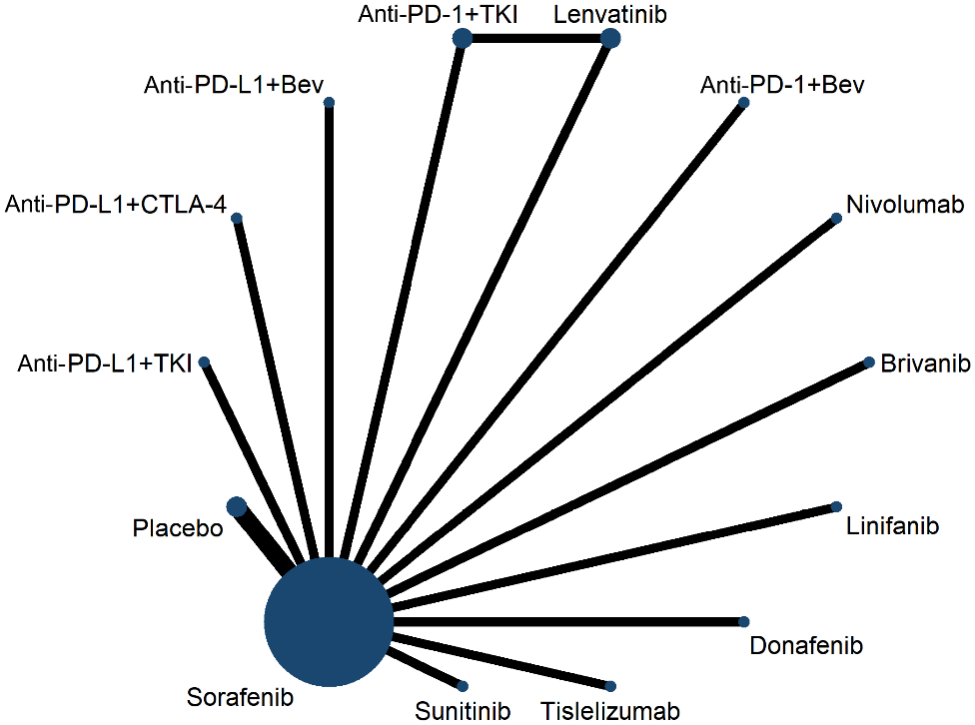
Network plot for overall survival for first-line trials. Anti-PD-1+Bev: programmed cell death 1 inhibitor combined with bevacizumab, anti-PD-L1+Bev: programmed death ligand 1 inhibitor combined with bevacizumab, anti-PD-1+TKI: programmed cell death 1 inhibitor combined with tyrosine kinase inhibitor, anti-PD-L1+TKI: programmed death ligand 1 inhibitor combined with tyrosine kinase inhibitor, anti-PD-L1+anti-CTLA-4: programmed death ligand 1 inhibitor combined with cytotoxic T cell lymphocyte antigen 4 inhibitor.

The first-line treatment studies included 15 papers containing a total of 10,912 patients (**Figure 2**), and two clinical trials comparing sorafenib with placebo (SHARP (5), Asia Pacific (24)). Five clinical trials comparing TKIs (lenvatinib, donafenib, brivanib, sunitinib, linifanib) with sorafenib were REFLECT (6), Qin 2021 (7), BRISK-FL (25), SUN1170 (26), and Cainap 2015 (27). Two clinical trials compared ICIs (nivolumab, tislelizumab) with sorafenib, CheckMate 459 (28), RATIONALE-301 (29). Two clinical trials compared ICIs (atezolizumab, sintilimab) in combination with bevacizumab versus sorafenib, IMbrave150 (13), ORIENT-32 (14), respectively. Two clinical trials compared ICIs in combination with TKIs (atezolizumab in combination with cabozantinib, camrelizumab combined with apatinib) with sorafenib, COSMIC-312 (15), SHR-1210-III-310 (17), respectively. The LEAP-002 (16) study compared lenvatinib in combination with pembrolizumab versus lenvatinib. HIMALAYA (18) compared tremelimumab in combination with durvalumab with sorafenib. All clinical trials were superior except for the REFLECT, Qin 2021, BRISK-FL, RATIONALE-301, and Cainap 2015 trials, which were noninferiority trials. Most clinical trials included patients with ECOG-PS scores of 0-2, Child-pugh classification of A, and BCLC stage B-C. Characteristics included in the RCTs are shown in **Supplementary Tables 2, 3**; **12–15**.

#### 3.1.2 NMA

NMA results showed that all treatment regimens except sunitinib (HR, 0.89; 95% CI, 0.71-1.13) significantly prolonged OS compared with placebo in patients with advanced HCC (**Table 1**). Compared with sorafenib (HR, 0.57; 95% CI, 0.43-0.75), lenvatinib (HR, 0.65; 95% CI, 0.48-0.88), donafenib (HR, 0.69; 95% CI, 0.5-0.95), nivolumab (HR, 0.67; 95% CI, 0.48-0.93), tislelizumab (HR, 0.67; 95% CI, 0.48-0.93), and PD-L1 inhibitors combined with TKI regimens (HR, 0.63; 95% CI, 0.43-0.93), PD-1 inhibitors combined with bevacizumab regimens showed OS benefit (**Table 1**). In the first-line treatment, The SUCRA value of PD-1 inhibitor combined with bevacizumab regimen (0.95) was the largest in OS, indicating that it most likely ranked first, followed by PD-L1 inhibitor plus bevacizumab regimen (0.94), PD-1 inhibitor plus TKI regimen (0.83), and PD-L1 inhibitor combined with CTLA-4 inhibitor regimen (0.70) (**Supplementary Table 4**). Compared with sorafenib (HR, 0.54; 95% CI, 0.46-0.64), lenvatinib (HR, 0.85; 95% CI, 0.74-0.98), donafenib (HR, 0.60; 95% CI, 0.47-0.76), nivolumab (HR, 0.58; 95% CI, 0.46-0.73), tislelizumab (HR, 0.49; 95% CI, 0.39-0.63), and PD-L1 inhibitor combined with CTLA-4 inhibitor regimens (HR, 0.60; 95% CI, 0.48-0.76), PD-1 inhibitor combined with TKI regimens had advantages in prolonging PFS (**Table 1**). The SUCRA value of PD-1 inhibitor combined with TKI regimen (0.93) was higher than that of PD-1 inhibitor combined with bevacizumab regimen (0.89) and PD-L1 inhibitor and CTLA-4 inhibitor regimen (0.46) in PFS (**Supplementary Table 5**). The PD-1 inhibitor combined with bevacizumab regimen and the PD-1 inhibitor combined with TKI regimen ranked the highest in terms of OS and PFS, with P values of 95% and 93%, respectively.

**Table 1:** Indirect comparisons of overall survival and progression-free survival among first-line treatments.

Treatment	Progression-free survival													
Overall Survival	**Anti-PD-1+Bev**	0.95 (0.69, 1.31)	1.03 (0.79, 1.35)	0.62 (0.48, 0.81)	0.89 (0.58, 1.36)	0.62 (0.47, 0.81)	0.6 (0.46, 0.79)	0.51 (0.39, 0.67)	0.88 (0.68, 1.13)	0.74 (0.56, 0.97)	0.55 (0.43, 0.71)	0.5 (0.39, 0.64)	0.56 (0.45, 0.69)	0.32 (0.24, 0.43)
	0.98 (0.64, 1.5)	**Anti-PD-L1+Bev**	1.09 (0.81, 1.46)	0.66 (0.49, 0.88)	0.94 (0.61, 1.45)	0.65 (0.48, 0.87)	0.63 (0.47, 0.85)	0.54 (0.4, 0.73)	0.93 (0.7, 1.22)	0.78 (0.58, 1.04)	0.58 (0.44, 0.77)	0.52 (0.4, 0.69)	0.59 (0.46, 0.75)	0.34 (0.25, 0.46)
	0.82 (0.59, 1.13)	0.83 (0.58, 1.19)	**Anti-PD-1+TKI**	0.6 (0.48, 0.76)	0.86 (0.57, 1.28)	0.6 (0.47, 0.76)	0.58 (0.46, 0.73)	0.49 (0.39, 0.63)	0.85 (0.74, 0.98)	0.71 (0.57, 0.9)	0.54 (0.43, 0.66)	0.48 (0.39, 0.59)	0.54 (0.46, 0.64)	0.31 (0.24, 0.4)
	0.73 (0.53, 1.02)	0.74 (0.52, 1.07)	0.89 (0.7, 1.14)	**Anti-PD-L1+Anti-CTLA-4**	1.43 (0.96, 2.12)	0.99 (0.78, 1.25)	0.97 (0.77, 1.22)	0.82 (0.64, 1.04)	1.41 (1.14, 1.74)	1.19 (0.94, 1.49)	0.89 (0.72, 1.1)	0.8 (0.65, 0.98)	0.9 (0.77, 1.05)	0.52 (0.4, 0.67)
	0.63 (0.43, 0.93)	0.65 (0.43, 0.97)	0.78 (0.57, 1.06)	0.87 (0.63, 1.19)	**Anti-PD-L1+TKI**	0.69 (0.46, 1.04)	0.68 (0.45, 1.01)	0.57 (0.38, 0.86)	0.99 (0.67, 1.46)	0.83 (0.56, 1.24)	0.62 (0.42, 0.92)	0.56 (0.38, 0.82)	0.63 (0.44, 0.91)	0.36 (0.24, 0.55)
	0.69 (0.5, 0.95)	0.7 (0.49, 1)	0.84 (0.66, 1.07)	0.94 (0.74, 1.2)	1.08 (0.79, 1.49)	**Donafenib**	0.98 (0.77, 1.24)	0.83 (0.64, 1.07)	1.42 (1.14, 1.78)	1.2 (0.94, 1.52)	0.9 (0.72, 1.12)	0.8 (0.64, 1)	0.91 (0.76, 1.08)	0.52 (0.4, 0.68)
	0.67 (0.48, 0.93)	0.68 (0.48, 0.98)	0.82 (0.65, 1.04)	0.92 (0.72, 1.17)	1.06 (0.77, 1.46)	0.98 (0.77, 1.25)	**Nivolumab**	0.85 (0.66, 1.08)	1.46 (1.17, 1.81)	1.22 (0.97, 1.55)	0.92 (0.74, 1.14)	0.82 (0.67, 1.02)	0.93 (0.79, 1.1)	0.54 (0.41, 0.69)
	0.67 (0.48, 0.93)	0.68 (0.47, 0.98)	0.82 (0.64, 1.05)	0.92 (0.72, 1.18)	1.06 (0.77, 1.46)	0.98 (0.76, 1.25)	1 (0.78, 1.28)	**Tislelizumab**	1.73 (1.37, 2.17)	1.45 (1.13, 1.85)	1.09 (0.86, 1.37)	0.97 (0.77, 1.22)	1.1 (0.91, 1.32)	0.63 (0.48, 0.83)
	0.65 (0.48, 0.88)	0.66 (0.47, 0.93)	0.79 (0.68, 0.92)	0.89 (0.71, 1.1)	1.02 (0.76, 1.38)	0.94 (0.76, 1.17)	0.96 (0.78, 1.2)	0.96 (0.77, 1.2)	**Lenvatinib**	0.84 (0.68, 1.04)	0.63 (0.52, 0.77)	0.56 (0.46, 0.69)	0.64 (0.56, 0.73)	0.37 (0.29, 0.47)
	0.54 (0.4, 0.75)	0.55 (0.39, 0.79)	0.67 (0.53, 0.84)	0.75 (0.59, 0.94)	0.86 (0.63, 1.17)	0.79 (0.63, 1)	0.81 (0.64, 1.02)	0.81 (0.64, 1.03)	0.84 (0.69, 1.03)	**Linifanib**	0.75 (0.6, 0.93)	0.67 (0.54, 0.83)	0.76 (0.64, 0.9)	0.44 (0.34, 0.57)
	0.54 (0.39, 0.73)	0.55 (0.39, 0.77)	0.66 (0.53, 0.82)	0.74 (0.59, 0.92)	0.85 (0.63, 1.15)	0.78 (0.63, 0.98)	0.8 (0.64, 1)	0.8 (0.64, 1)	0.83 (0.69, 1)	0.99 (0.8, 1.21)	**Brivanib**	0.89 (0.74, 1.09)	1.01 (0.88, 1.16)	0.58 (0.46, 0.74)
	0.44 (0.32, 0.6)	0.45 (0.32, 0.63)	0.54 (0.43, 0.67)	0.6 (0.48, 0.75)	0.69 (0.51, 0.94)	0.64 (0.51, 0.8)	0.65 (0.52, 0.82)	0.65 (0.52, 0.82)	0.68 (0.56, 0.82)	0.81 (0.65, 0.99)	0.82 (0.67, 0.99)	**Sunitinib**	1.13 (0.99, 1.29)	0.65 (0.51, 0.83)
	0.57 (0.43, 0.75)	0.58 (0.42, 0.8)	0.7 (0.59, 0.82)	0.78 (0.66, 0.93)	0.9 (0.69, 1.18)	0.83 (0.7, 0.99)	0.85 (0.71, 1.01)	0.85 (0.71, 1.02)	0.88 (0.77, 1.01)	1.05 (0.9, 1.22)	1.06 (0.93, 1.21)	1.3 (1.13, 1.5)	**Sorafenib**	0.58 (0.47, 0.7)
	0.39 (0.28, 0.55)	0.4 (0.28, 0.57)	0.48 (0.37, 0.61)	0.54 (0.42, 0.69)	0.62 (0.45, 0.86)	0.57 (0.44, 0.73)	0.58 (0.45, 0.75)	0.58 (0.45, 0.75)	0.6 (0.48, 0.76)	0.72 (0.56, 0.91)	0.73 (0.58, 0.92)	0.89 (0.71, 1.13)	0.69 (0.57, 0.83)	**Placebo**

Pooled hazard ratio (HR) and 95% CI for overall survival (OS) and progression-free survival (PFS) in the overall population. Anti-PD-1+Bev: programmed cell death 1 inhibitor combined with bevacizumab, anti-PD-L1+Bev: programmed death ligand 1 inhibitor combined with bevacizumab, anti-PD-1+TKI: programmed cell death 1 inhibitor combined with tyrosine kinase inhibitor, anti-PD-L1+TKI: programmed death ligand 1 inhibitor combined with tyrosine kinase inhibitor, anti-PD-L1+anti-CTLA-4: programmed death ligand 1 inhibitor combined with cytotoxic T cell lymphocyte antigen 4 inhibitor.

All 15 included papers reported ORR results, and the NMA showed that compared with sorafenib, PD-1 inhibitors combined with bevacizumab (HR, 6.36; 95% CI, 1.34-32.54), and PD-L1 inhibitors combined with CTLA-4 inhibitor regimens (HR, 4.69; 95% CI, 1.06-21.28) demonstrated ORR benefit (**Table 2**). The SUCRA value for PD-1 inhibitor plus bevacizumab regimen (0.86) was the largest in ORR, followed by PD-1 inhibitor combined with TKI regimen (0.85), and PD-L1 inhibitor combined with CTLA-4 inhibitor regimen (0.78) (**Supplementary Table 6**). Thirteen of the included papers reported grade 3 or higher adverse reaction rates (SHARP, Asia Pacific did not report and therefore were not included in the analysis). In terms of grade 3 or more adverse reactions, the first three lowest rates of adverse reactions were nivolumab (0.9), tislelizumab (0.78) and PD-L1 inhibitors combined with CTLA-4 inhibitor regimen (0.78) (**Table 3**, **Supplementary Table 7**).

**Table 2:** Indirect comparisons of objective response rate among first-line treatments.

Anti-PD-1+Bev													
2.27 (0.27, 20.48)	**Anti-PD-L1+Bev**												
1.15 (0.16, 9.11)	0.52 (0.07, 3.55)	**Anti-PD-1+TKI**											
1.36 (0.15, 12.25)	0.6 (0.07, 4.95)	1.16 (0.17, 7.89)	**Anti-PD-L1+Anti-CTLA-4**										
1.88 (0.2, 19.06)	0.84 (0.09, 7.34)	1.62 (0.21, 12.25)	1.38 (0.16, 12.45)	**Anti-PD-L1+TKI**									
3.63 (0.39, 36.39)	1.61 (0.18, 14.12)	3.1 (0.42, 23.57)	2.67 (0.3, 24.1)	1.91 (0.21, 18.28)	**Donafenib**								
2.62 (0.31, 24.84)	1.18 (0.14, 9.85)	2.27 (0.34, 15.74)	1.95 (0.24, 16.66)	1.39 (0.16, 12.32)	0.73 (0.08, 6.59)	**Nivolumab**							
2.14 (0.25, 19.82)	0.95 (0.12, 7.97)	1.84 (0.27, 12.45)	1.56 (0.19, 13.27)	1.14 (0.13, 10.01)	0.59 (0.07, 5.32)	0.81 (0.1, 6.78)	**Tislelizumab**						
1.9 (0.27, 14.67)	0.85 (0.12, 5.73)	1.65 (0.49, 5.47)	1.41 (0.21, 9.88)	1.01 (0.14, 7.53)	0.53 (0.07, 3.92)	0.73 (0.1, 4.95)	0.89 (0.13, 6)	**Lenvatinib**					
3.68 (0.43, 31.64)	1.65 (0.2, 12.85)	3.17 (0.45, 21.31)	2.7 (0.33, 22.56)	1.96 (0.22, 16.74)	1.02 (0.11, 8.77)	1.4 (0.16, 10.95)	1.73 (0.21, 13.73)	1.93 (0.28, 12.65)	**Linifanib**				
4.62 (0.53, 41.91)	2.06 (0.25, 16.74)	4 (0.59, 27.6)	3.41 (0.43, 28.25)	2.47 (0.29, 21.34)	1.28 (0.14, 11.57)	1.75 (0.21, 13.88)	2.18 (0.27, 17.93)	2.43 (0.35, 16.46)	1.25 (0.16, 10.43)	**Brivanib**			
5.84 (0.65, 52.07)	2.56 (0.3, 21.17)	5.01 (0.72, 33.49)	4.25 (0.52, 35.1)	3.1 (0.34, 26.34)	1.6 (0.17, 14.06)	2.2 (0.26, 18.04)	2.7 (0.32, 22.2)	3.05 (0.44, 20.1)	1.55 (0.2, 13.16)	1.26 (0.15, 10.04)	**Sunitinib**		
6.36 (1.34, 32.54)	2.85 (0.62, 12.37)	5.51 (1.62, 18.45)	4.69 (1.06, 21.28)	3.4 (0.69, 16.85)	1.77 (0.35, 8.77)	2.41 (0.53, 10.52)	3.01 (0.66, 13.31)	3.35 (0.99, 11.09)	1.74 (0.4, 7.53)	1.38 (0.3, 6.02)	1.1 (0.25, 4.96)	**Sorafenib**	
15.83 (1.92, 157.78)	7.13 (0.9, 61.3)	13.85 (2.09, 102.29)	11.8 (1.5, 108.59)	8.45 (0.99, 79.61)	4.39 (0.5, 42.63)	6.05 (0.77, 53.43)	7.5 (0.94, 65.73)	8.34 (1.29, 61.25)	4.33 (0.57, 37.91)	3.45 (0.44, 30.32)	2.74 (0.36, 24.6)	2.49 (0.57, 12.65)	**Placebo**

Pooled relative risk (RR) and 95% CI for objective response rate (ORR) in the overall population. Anti-PD-1+Bev: programmed cell death 1 inhibitor combined with bevacizumab, anti-PD-L1+Bev: programmed death ligand 1 inhibitor combined with bevacizumab, anti-PD-1+TKI: programmed cell death 1 inhibitor combined with tyrosine kinase inhibitor, anti-PD-L1+TKI: programmed death ligand 1 inhibitor combined with tyrosine kinase inhibitor, anti-PD-L1+anti-CTLA-4: programmed death ligand 1 inhibitor combined with cyto-toxic T cell lymphocyte antigen 4 inhibitor.

**Table 3:** Indirect comparisons of adverse events of grade 3 or higher among first-line treatments.

Anti-PD-1+Bev												
1.31 (0.13, 13.27)	**Anti-PD-L1+Bev**											
0.46 (0.05, 3.83)	0.35 (0.04, 3)	**Anti-PD-1+TKI**										
2.06 (0.2, 21.32)	1.58 (0.16, 16.29)	4.52 (0.55, 38.59)	**Anti-PD-L1+Anti-CTLA-4**									
0.56 (0.06, 5.86)	0.43 (0.04, 4.51)	1.24 (0.15, 10.77)	0.27 (0.03, 2.89)	**Anti-PD-L1+TKI**								
2.05 (0.2, 20.92)	1.56 (0.15, 16.07)	4.49 (0.54, 38.28)	0.99 (0.1, 10.09)	3.63 (0.35, 36.82)	**Donafenib**							
4.49 (0.44, 46.43)	3.42 (0.34, 35.87)	9.81 (1.18, 85.78)	2.18 (0.21, 22.13)	7.98 (0.78, 79.26)	2.19 (0.21, 22.81)	**Nivolumab**						
2.66 (0.26, 27.34)	2.03 (0.2, 20.55)	5.81 (0.71, 49.33)	1.29 (0.13, 13.11)	4.71 (0.46, 46.31)	1.3 (0.13, 13.14)	0.59 (0.06, 5.96)	**Tislelizumab**					
0.74 (0.09, 6.28)	0.56 (0.07, 4.78)	1.61 (0.42, 6.22)	0.36 (0.04, 2.93)	1.3 (0.15, 10.87)	0.36 (0.04, 3)	0.16 (0.02, 1.36)	0.28 (0.03, 2.29)	**Lenvatinib**				
0.68 (0.07, 6.99)	0.51 (0.05, 5.29)	1.47 (0.18, 12.77)	0.33 (0.03, 3.34)	1.2 (0.11, 12.12)	0.33 (0.03, 3.37)	0.15 (0.01, 1.53)	0.25 (0.03, 2.61)	0.91 (0.11, 7.79)	**Linifanib**			
1.21 (0.12, 12.61)	0.92 (0.09, 9.51)	2.66 (0.32, 22.56)	0.59 (0.06, 6)	2.15 (0.21, 21.78)	0.6 (0.06, 6.02)	0.27 (0.03, 2.7)	0.46 (0.04, 4.62)	1.65 (0.2, 13.83)	1.8 (0.17, 17.95)	**Brivanib**		
0.82 (0.08, 8.42)	0.63 (0.06, 6.42)	1.8 (0.22, 15.53)	0.4 (0.04, 4.2)	1.46 (0.14, 14.71)	0.4 (0.04, 4.19)	0.18 (0.02, 1.91)	0.31 (0.03, 3.17)	1.12 (0.14, 9.5)	1.22 (0.12, 12.6)	0.68 (0.07, 7.02)	**Sunitinib**	
1.32 (0.25, 6.83)	1 (0.19, 5.32)	2.88 (0.75, 11.38)	0.64 (0.12, 3.3)	2.33 (0.45, 11.91)	0.64 (0.13, 3.33)	0.29 (0.06, 1.53)	0.5 (0.1, 2.53)	1.79 (0.47, 6.91)	1.95 (0.38, 10.06)	1.09 (0.21, 5.64)	1.6 (0.31, 8.24)	**Sorafenib**

Pooled relative risk (RR) and 95% CI for adverse events of grade 3 or higher (≥3AEs) in the overall population. Anti-PD-1+Bev: programmed cell death 1 inhibitor combined with bevacizumab, anti-PD-L1+Bev: programmed death ligand 1 inhibitor combined with bevacizumab, anti-PD-1+TKI: programmed cell death 1 inhibitor combined with tyrosine kinase inhibitor, anti-PD-L1+TKI: programmed death ligand 1 inhibitor combined with tyrosine kinase inhibitor, anti-PD-L1+anti-CTLA-4: programmed death ligand 1 inhibitor combined with cytotoxic T cell lymphocyte antigen 4 inhibitor.

#### 3.1.3 Risk of bias

The risk of bias results from the first line of 15 publications evaluated according to the Cochrane Risk of Bias Assessment Tool showed that all trials reported a “low risk of bias” in at least 5 out of the 7 domains of interest. The treatment arms of the Cainap 2015, CheckMate 459, COSMIC-312, HIMALAYA, IMbrave 150, ORIENT-32, Quin 2021, RE-FLECT and SUN1170 studies were not found to be explicitly blinded to subjects and personnel in the treatment arms. Cainap 2015 and SUN1170 were not found to be explicitly blinded in the outcome assessment part (**Supplementary Figure 1**).

### 3.2 Second-line treatments

#### 3.2.1 Baseline characteristics

**Figure 3:**
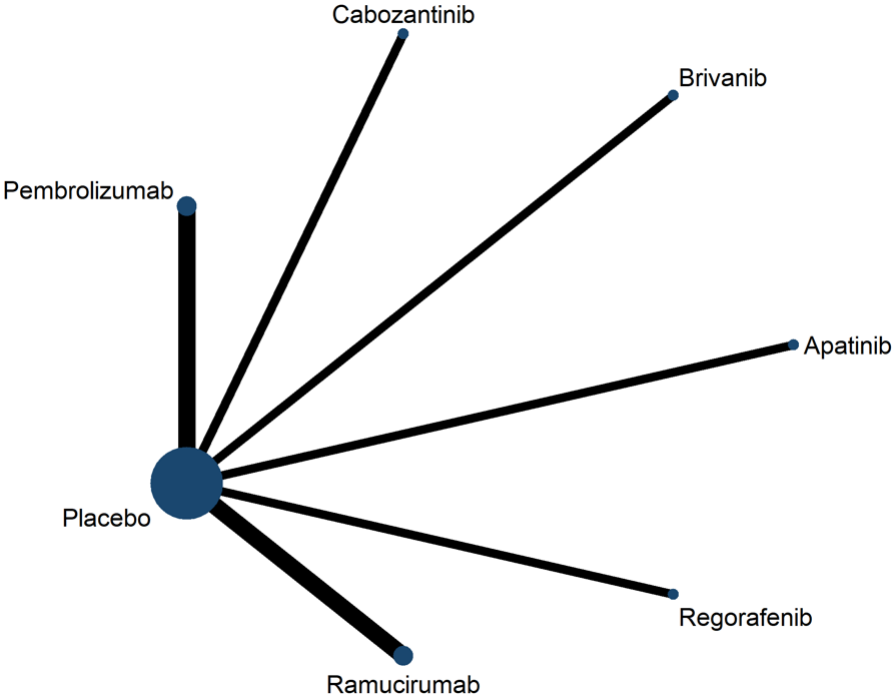
Network plot for overall survival for second-line trials.

Second-line treatment studies included eight publications containing a total of 3,791 patients, six clinical trials comparing antiangiogenic drugs (apatinib, brivanib, cabozantinib, regorafenib, ramucirumab) versus placebo, and two clinical trials com-paring ICIs (pembrolizumab) versus placebo (**Figure 3**). Most of the clinical trials enrolled patients with ECOG-PS score 0-2, Child-pugh classification A, BCLC stage B-C. Characteristics included in the RCT are shown in **Supplementary Table 2**.

#### 3.2.2 NMA

NMA results showed that all treatment regimens prolonged PFS compared with placebo in patients with advanced HCC (**Table 4**), however this only translated into OS benefit in patients with advanced HCC with regorafenib (HR, 0.63; 95% CI, 0.5-0.79), cabozantinib (HR, 0.76; 95% CI, 0.63-0.92), ramucirumab (HR, 0.82; 95% CI, 0.7-0.96), and pembrolizumab (HR, 0.79; 95% CI, 0.67-0.93). Cabozantinib, regorafenib, and apatinib significantly prolonged PFS in patients with advanced HCC compared with pembrolizumab. Cabozantinib and regorafenib were more advantageous in prolonging PFS compared with ramucirumab. Regorafenib and cabozantinib ranked highest in OS and PFS with P values of 94.65% and 87.36%, respectively (**Supplementary Tables 8, 9**).

**Table 4:** Indirect comparisons of overall survival and progression-free survival among second-line treatment.

Treatment	Progression-free survival						
Overall Survival	**Brivanib**	1.22 (0.9, 1.65)	1.27 (0.9, 1.8)	1.19 (0.81, 1.74)	0.77 (0.55, 1.07)	0.97 (0.7, 1.36)	0.56 (0.42, 0.75)
	1.41 (1, 1.99)	**Regorafenib**	1.05 (0.86, 1.27)	0.98 (0.76, 1.26)	0.63 (0.53, 0.75)	0.8 (0.67, 0.95)	0.46 (0.43, 0.49)
	1.17 (0.85, 1.61)	0.83 (0.61, 1.12)	**Cabozantinib**	0.93 (0.69, 1.27)	0.6 (0.47, 0.77)	0.77 (0.6, 0.97)	0.44 (0.37, 0.53)
	1.13 (0.8, 1.61)	0.8 (0.57, 1.12)	0.97 (0.71, 1.32)	**Apatinib**	0.65 (0.48, 0.86)	0.82 (0.61, 1.1)	0.47 (0.37, 0.6)
	1.13 (0.83, 1.54)	0.8 (0.6, 1.06)	0.97 (0.75, 1.25)	1 (0.75, 1.34)	**Pembrolizumab**	1.27 (1.02, 1.58)	0.73 (0.62, 0.85)
	1.09 (0.81, 1.47)	0.77 (0.58, 1.01)	0.93 (0.72, 1.19)	0.96 (0.72, 1.28)	0.96 (0.76, 1.21)	**Ramucirumab**	0.58 (0.49, 0.67)
	0.89 (0.69, 1.15)	0.63 (0.5, 0.79)	0.76 (0.63, 0.92)	0.79 (0.62, 1)	0.79 (0.67, 0.93)	0.82 (0.7, 0.96)	**Placebo**

Pooled hazard ratio (HR) and 95% CI for overall survival (OS) and progression-free survival (PFS) in the overall population.

ORR results were reported in the eight included papers, and the NMA showed that ramucirumab (HR, 9.19; 95% CI, 1.35-83.57), and pembrolizumab (HR, 7.48; 95% CI, 1.47-51.13) demonstrated an ORR benefit compared with placebo (**Table 5**). Cabozantinib ranked the highest on ORR with a P value of 70.42%. Six of the included papers reported grade 3 or higher adverse reaction rates (REACH, REACH-2 were not reported and therefore not included in the analysis), and apatinib and brivanib had the highest incidence of ≥3 AEs among all treatment modalities, with p-values of 15.14% and 32.51%, respectively (**Table 6**, **Supplementary Tables 10, 11**).

**Table 5:** Indirect comparisons of objective response rate among second-line treatments.

Brivanib						
3.01 (0.09, 121.21)	**Regorafenib**					
0.63 (0.01, 35.05)	0.21 (0, 8.38)	**Cabozantinib**				
0.92 (0.02, 40.95)	0.3 (0.01, 10.17)	1.44 (0.03, 144.93)	**Apatinib**			
1.13 (0.04, 30.13)	0.38 (0.02, 6.66)	1.82 (0.06, 111.48)	1.23 (0.05, 32.58)	**Pembrolizumab**		
0.94 (0.03, 28.85)	0.31 (0.01, 6.38)	1.46 (0.04, 112.5)	1.03 (0.04, 30.89)	0.81 (0.05, 12.82)	**Ramucirumab**	
8.57 (0.66, 154.76)	2.87 (0.27, 31.07)	13.61 (0.83, 647.19)	9.36 (0.75, 163.37)	7.48 (1.47, 51.13)	9.19 (1.35, 83.57)	**Placebo**

Pooled relative risk (RR) and 95% CI for objective response rate (ORR) in the overall population.

**Table 6:** Indirect comparisons of adverse events of grade 3 or higher among second-line treatments.

Brivanib					
1.36 (0.02, 85.33)	**Regorafenib**				
1.87 (0.03, 109.88)	1.37 (0.02, 84.09)	**Cabozantinib**			
0.47 (0.01, 29.6)	0.35 (0.01, 21.96)	0.25 (0, 15.54)	**Apatinib**		
3.98 (0.1, 127.52)	2.95 (0.08, 95.2)	2.14 (0.06, 70.01)	8.39 (0.21, 284.24)	**Pembrolizumab**	
6.91 (0.36, 125.12)	5.1 (0.27, 93.15)	3.69 (0.2, 66.83)	14.53 (0.78, 268.85)	1.74 (0.23, 14.9)	**Placebo**

Pooled relative risk (RR) AND 95% CI for adverse events of grade 3 or higher (≥3AEs) in the overall population.

#### 3.2.3 Risk of bias

The risk of bias was evaluated according to the Cochrane Risk of Bias Assessment Tool for the second-line 8 literature, and these trials were considered to be at low risk of bias. The CELESTIAL, REACH, and RESORCE studies did not describe the blinding of the outcome assessment in detail. The REACH-2 study may have lacked the reporting of some of the outcomes (**Supplementary Figure 2**).

## 4 Discussion

There is a wide variety of treatment options for advanced HCC, including targeted agents, immune checkpoint inhibitors, and targeted agents plus immune checkpoint inhibitors (30). However, head-to-head direct comparisons of the efficacy and safety of most therapeutic agents are lacking. In order to provide oncologists with more direct evidence, we conducted the largest NMA to date exploring a variety of systemic therapeutic regimens in first- and second-line treatment studies of advanced HCC, comparing their efficacy and safety.

The NMA results suggest that the PD-1 inhibitor in combination with bevacizumab regimen showed OS and ORR benefit and the PD-1 inhibitor in combination with TKI regimen showed PFS benefit compared to placebo, sorafenib, lenvatinib, and nivolumab. On the other hand, in the refractory HCC population, regorafenib and cabozantinib showed OS and PFS, ORR benefit.

In the first-line treatment of advanced HCC, ICIs combined with bevacizumab regimen and ICIs combined with TKI regimen are the two most effective first-line treatment regimens. The development of HCC involves many cell signaling pathways and cytokines (31). Vascular endothelial growth factor (VEGF) plays an important role in this process (32). Many studies have demonstrated that the combination of anti-angiogenic drugs and immune checkpoint inhibitors may have a synergistic effect in promoting tumor vascular normalization and stimulating immune activation. Normalization of tumor vasculature can promote the aggregation of immune cells as well as enhance the immune function, while the activation of immune cells can, in turn, promote vascular normalization, so the combination of the two can theoretically play the effect of 1 + 1>2 (33, 34). This is consistent with the results of this study. The RCTs involved in the ICIs in combination with bevacizumab regimen include the IMbrave150 and ORIENT-32 clinical trials, and the RCTs involved in ICIs in combination with TKIs include the COSMIC-312, LEAP-002, and the SHR-1210-III-310 studies. A phase III trial suggests that first-line treatment of advanced HCC with sintilimab in combination with bevacizumab (ORI-ENT-32) is superior to sorafenib in reducing the risk of death in HCC patients by 43% (14).This is consistent with the results of the present study, which ranked sintilimab in combination with bevacizumab the highest in terms of prolonging OS in patients with advanced HCC compared with all other treatments, including sorafenib, lenvatinib, donafenib, nivolumab, and tislelizumab. Therefore, ICIs in combination with bevacizumab regimens are now considered the standard of care in the first-line treatment of most patients with advanced HCC. However, in terms of PFS, the phase III clinical trial SHR-1210-III-310 suggested that camrelizumab in combination with apatinib significantly prolonged PFS over sorafenib in patients with advanced HCC (HR, 0.52; 95% CI, 0.41-0.65) (17), so in terms of prolongation of PFS in the present study, the PD-1 inhibitor in com-bination with the TKI regimen ranked the highest. However, it is worth noting that clinical trials involving PD-1/PD-L1 + TKI combinations presented mixed results. COSMIC-312 (atilizumab in combination with cabozantinib) (15) and LEAP-002 (pembrolizumab in combination with lenvatinib) (16) did not reach their primary study endpoints. Although optimistic efficacy data were reported in the phase Ib/II trial (35), the results of the phase III clinical trial triggered more controversy. Therefore, whether there is an intrinsic immune difference between large molecule monoclonal antibodies (anti-VEGF) and small molecule multi-kinase inhibitor (TKIs) is still the subject of debate, and more clinical trial studies are needed in the future. Although ICIs combined with TKIs bring more survival benefits, they are accompanied by more toxic side effects, which also deserve our attention, so when choosing a treatment regimen in the clinic, we should not only focus on the efficacy, but also consider the safety of the regimen.

Lenvatinib and sorafenib remain preferred options for patients who are intolerant of, or have contraindications to, immunotherapy. Notably the only treatment regimen that did not involve the use of an anti-angiogenic drug and achieved OS improvement was the combination of durvalumab with tremelimumab. This combination was approved by the FDA (36) with fewer toxicities and higher survival benefit, making it a potential alter-native to atezolizumab in combination with bevacizumab in first-line therapy, and therefore a preferred option in patients with absolute contraindications to VEGF inhibitors.

In second-line treatment of advanced HCC, most clinical trials enrolled patients with progression/intolerance to sorafenib, and the results of our analysis showed that regorafenib had the highest scores in terms of OS. Cabozantinib had the highest scores in terms of PFS and ORR, but the incidence of ≥3AE was higher for regorafenib than for cabozantinib. However, it is worth noting that the CELESTIAL phase III trial (cabozantinib vs placebo), which included a population that allowed for the use of multiple frontline therapies and did not strictly limit sorafenib tolerance, included 2% of patients who had been treated with regorafenib, and 3% of patients who had been previously treated with ICIs (9). Therefore, for patients using ICIs in combination with bevacizumab as first-line treatment, the choice of regimen for second-line treatment after progression remains to be further investigated. The KEYNOTE-240 phase III clinical trial (pembrolizumab vs placebo) failed to reach its prespecified study endpoints, which was later vindicated by KEYNOTE-394, and while some of the reasons for the treatment’s failure, and success, have been discussed, they ultimately remain unclear. In recent years, immune or targeted drug therapies are changing the treatment landscape for advanced HCC, but systemic therapy is also challenged by primary and secondary resistance. Targeted treatments plus ICIs therapies as well as sequential therapies remain controversial at present. The activity of TKIs (e.g., sorafenib, lenvatinib, etc.) and anti-VEGF monoclonal antibody (ramucirumab) after progression to bevacizumab therapy, and second-line use of pembrolizumab after exposure to first-line ICIs still needs to be further investigated in a large number of clinical trials.

This study has several limitations. First, given that direct evidence is more important than indirect evidence, lack of direct evidence in some of the experiments in this study is due to the use of sorafenib in control group of most first-line clinical trials and the use of placebo in control group of second-line clinical trials, limiting the power of this analysis. Second, not all included studies had large sample sizes, which may have weakened the statistical power of the NMA. Most of the original studies in the first-line treatment of advanced HCC used TKI monotherapy as a control group, and there is a lack of evidence-based medical evidence comparing the advantages and disadvantages of ICIs plus bevacizumab and ICIs plus TKI, as well as the efficacy and safety of dual immunotherapy compared with these two regimens, which needs to be verified by more clinical trials. It is expected that future clinical trials will be able to do subgroup analyses of this population to determine whether dual immunotherapy is more beneficial for patients with high PD-1/PD-L1 expression. In the second-line treatment of advanced HCC, does the treatment regimen prior to the second line affect the efficacy of the later treatment regimen, and is there any benefit in continuing immunotherapy in the second line after applying immunotherapy combined with anti-vascular therapy in the first line? Is combination or sequential therapy more advantageous for immunological and targeted therapies? The direction of future clinical trials should evaluate the above aspects to provide guidance for more comprehensive development of precise individualized treatment plans.

## 5 Conclusions

In recent years, the treatment landscape of advanced unresectable HCC has changed significantly. By analyzing various clinical treatment regimens for advanced HCC, ICIs plus bevacizumab, ICIs plus TKI, and dual immunotherapy in first-line treatment all significantly improved OS in patients with advanced HCC.ICIs plus bevacizumab was associated with better OS, ORR, and ICIs plus TKI was associated with better PFS, and higher incidence of grade 3 or higher adverse events. Notably dual immunotherapy has a better safety profile and is a preferred option for patients with absolute contraindications to VEGF inhibitors. In second-line treatment of advanced HCC, regorafenib and cabozantinib significantly improved OS in patients with advanced HCC. Regorafenib was more prolonged OS in patients with HCC, and cabozantinib was associated with the best PFS, ORR. The incidence of grade 3 or higher adverse reactions was higher with regorafenib. In conclusion, our NMA provides a new perspective on the role of first- and second-line therapeutic regimens in advanced HCC to provide a reference for the selection of clinical therapeutic regimens.

## Data availability statement

The original contributions presented in the study are included in the article/**Supplementary Material**. Further inquiries can be directed to the corresponding author.

## Author contributions

DW: Data curation, Writing – original draft, Writing – review & editing. BJ: Writing – review & editing. MJ: Writing – review & editing. HTZ: Software, Writing – review & editing. HZ: Methodology, Writing – review & editing. JZ: Conceptualization, Supervision, Writing – review & editing.
