# Supplementary material for: Comparative efficacy and safety of systemic therapy for advanced hepatocellular carcinoma: a systematic review and network meta-analysis
Source: Front Oncol. 2023 Dec 6;13:1274754. doi: 10.3389/fonc.2023.1274754 (PMC10730675; doi:10.3389/fonc.2023.1274754)
Supplement: Supplementary file 1 [file DataSheet_1.pdf]

Supplementary Material

Supplementary Tables

Database	Period	Search Steps
PubMed	12,2022	("adult liver cancer*"[Title/Abstract] OR "carcinoma of the liver"[Title/Abstract] OR "hepatic carcinoma"[Title/Abstract] OR "hepatic cell carcinoma"[Title/Abstract] OR "hepato carcinoma"[Title/Abstract] OR "hepato cellular carcinoma"[Title/Abstract] OR "hepatocarcinoma"[Title/Abstract] OR "hepatocellular carcinoma*"[Title/Abstract] OR "hepatocellular carcinomata"[Title/Abstract] OR "hepatocyte carcinoma"[Title/Abstract] OR "hepatoma*"[Title/Abstract] OR "hepatomata"[Title/Abstract] OR "hepatomatous"[Title/Abstract] OR "liver carcinoma"[Title/Abstract] OR "Liver Cell Carcinoma"[Title/Abstract] OR "Liver Cell Carcinomas"[Title/Abstract] OR "malignant hepatoma"[Title/Abstract] OR "primary liver carcinoma"[Title/Abstract] OR "carcinoma, hepatocellular"[MeSH Terms]) AND ("blockade pd 1 pd l1"[Title/Abstract] OR "checkpoint blockade immune"[Title/Abstract] OR "checkpoint blockers immune"[Title/Abstract] OR "checkpoint inhibition immune"[Title/Abstract] OR "checkpoint inhibitor immune"[Title/Abstract] OR "checkpoint inhibitors immune"[Title/Abstract] OR "ctla 4 inhibitor*"[Title/Abstract] OR "Cytotoxic T Lymphocyte Associated Protein 4 Inhibitor"[Title/Abstract] OR "Cytotoxic T Lymphocyte Associated Protein 4 Inhibitors"[Title/Abstract] OR "Immune Checkpoint Blockade"[Title/Abstract] OR "immune checkpoint blocker*"[Title/Abstract] OR "Immune Checkpoint Inhibition"[Title/Abstract] OR "Immune Checkpoint Inhibitor"[Title/Abstract] OR "Immune Checkpoint Inhibitors"[Title/Abstract] OR "inhibitor pd 1"[Title/Abstract] OR "pd 1 inhibitor*"[Title/Abstract] OR "PD 1 PD L1 Blockade"[Title/Abstract] OR "pd l1 inhibitor*"[Title/Abstract] OR "programmed cell death protein 1 inhibitor*"[Title/Abstract] OR "Programmed Death Ligand 1

		Inhibitors"[Title/Abstract] OR "Immune Checkpoint Inhibitors"[MeSH Terms] OR ("Nivolumab"[Title/Abstract] OR "Atezolizumab"[Title/Abstract] OR "Ipilimumab"[Title/Abstract] OR "Pembrolizumab"[Title/Abstract] OR "Camrelizumab"[Title/Abstract] OR "Cemiplimab"[Title/Abstract] OR "Durvalumab"[Title/Abstract] OR "Sintilimab"[Title/Abstract]) OR ("Tyrosine kinase inhibitors"[Title/Abstract] OR "TKI"[Title/Abstract] OR "Multikinase inhibitor"[Title/Abstract] OR "Sorafenib"[Title/Abstract] OR "Lenvatinib"[Title/Abstract] OR "Regorafenib"[Title/Abstract] OR "Cabozantinib"[Title/Abstract] OR "Gefitinib"[Title/Abstract] OR "Brivanib"[Title/Abstract] OR "Dovitinib"[Title/Abstract] OR "Erlotinib"[Title/Abstract] OR "Everolimus"[Title/Abstract] OR "Linifanib"[Title/Abstract] OR "Nintedanib"[Title/Abstract] OR "Orantinib"[Title/Abstract] OR "Vandetanib"[Title/Abstract] OR "Bevacizumab"[Title/Abstract])) AND ("randomized"[Title/Abstract] OR "RCT"[Title/Abstract]) AND "Advanced"[Title/Abstract] (342)
Embase	12,2022	1. 'adult liver cancer*':ti,ab,kw OR 'carcinoma in the liver':ti,ab,kw OR 'carcinoma of the liver':ti,ab,kw OR 'hepatic carcinoma':ti,ab,kw OR 'hepatic cell carcinoma':ti,ab,kw OR 'hepato carcinoma':ti,ab,kw OR 'hepato cellular carcinoma':ti,ab,kw OR 'hepatocarcinoma':ti,ab,kw OR 'hepatocellular carcinoma*':ti,ab,kw OR 'hepatocellular carcinomata':ti,ab,kw OR 'hepatocyte carcinoma':ti,ab,kw OR 'hepatocytic carcinoma':ti,ab,kw OR 'hepatoma*':ti,ab,kw OR 'hepatomata':ti,ab,kw OR 'hepatomatous':ti,ab,kw OR 'liver carcinoma':ti,ab,kw OR 'liver carcinoma rupture':ti,ab,kw OR 'liver cell carcinoma':ti,ab,kw OR 'liver cell carcinoma, adult':ti,ab,kw OR 'liver cell carcinomas':ti,ab,kw OR 'malignant hepatoma':ti,ab,kw OR 'primary liver carcinoma':ti,ab,kw<br>2. 'liver cell carcinoma'/exp<br>3. 'blockade, pd 1 pd l1':ti,ab,kw OR 'checkpoint blockade, immune':ti,ab,kw OR 'checkpoint blockers, immune':ti,ab,kw OR 'checkpoint inhibition, immune':ti,ab,kw OR 'checkpoint inhibitor, immune':ti,ab,kw OR 'checkpoint inhibitors, immune':ti,ab,kw OR 'ctla 4 inhibitor*':ti,ab,kw OR 'cytotoxic t lymphocyte associated protein 4 inhibitor':ti,ab,kw OR 'cytotoxic t lymphocyte associated protein 4 inhibitors':ti,ab,kw

		OR 'immune checkpoint blockade':ti,ab,kw OR 'immune checkpoint blocker*':ti,ab,kw OR 'immune checkpoint inhibition':ti,ab,kw OR 'immune checkpoint inhibitor':ti,ab,kw OR 'immune checkpoint inhibitors':ti,ab,kw OR 'inhibitor, pd 1':ti,ab,kw OR 'pd 1 inhibitor*':ti,ab,kw OR 'pd 1 pd l1 blockade':ti,ab,kw OR 'pd l1 inhibitor*':ti,ab,kw OR 'programmed cell death protein 1 inhibitor*':ti,ab,kw OR 'programmed death ligand 1 inhibitors':ti,ab,kw<br>4. 'immune checkpoint inhibitor'/exp<br>5. 'nivolumab':ti,ab,kw OR 'atezolizumab':ti,ab,kw OR 'ipilimumab':ti,ab,kw OR 'pembrolizumab':ti,ab,kw OR 'camrelizumab':ti,ab,kw OR 'cemiplimab':ti,ab,kw OR 'durvalumab':ti,ab,kw OR 'sintilimab':ti,ab,kw<br>6. 'tyrosine kinase inhibitors':ti,ab,kw OR 'tki':ti,ab,kw OR 'multikinase inhibitor':ti,ab,kw OR 'sorafenib':ti,ab,kw OR 'lenvatinib':ti,ab,kw OR 'regorafenib':ti,ab,kw OR 'cabozantinib':ti,ab,kw OR 'gefitinib':ti,ab,kw OR 'brivanib':ti,ab,kw OR 'dovitinib':ti,ab,kw OR 'erlotinib':ti,ab,kw OR 'everolimus':ti,ab,kw OR 'linifanib':ti,ab,kw OR 'nintedanib':ti,ab,kw OR 'orantinib':ti,ab,kw OR 'slunitinib':ti,ab,kw OR 'vandetanib':ti,ab,kw OR 'bevacizumab':ti,ab,kw<br>7. 'randomized':ti,ab,kw OR 'rct':ti,ab,kw<br>8. 'advanced':ti,ab,kw<br>9. (#1 OR #2) AND (#3 OR #4 OR #5 OR #6) AND #7 AND #8 (880)
Cochrane	12,2022	1. ('Adult Liver Cancer*' OR 'carcinoma in the liver' OR 'carcinoma of the liver' OR 'hepatic carcinoma' OR 'hepatic cell carcinoma' OR 'hepato carcinoma' OR 'hepato cellular carcinoma' OR 'hepatocarcinoma' OR 'Hepatocellular Carcinoma*' OR 'hepatocellular carcinomata' OR 'hepatocyte carcinoma' OR 'hepatocytic carcinoma' OR 'Hepatoma*' OR 'hepatomata' OR 'hepatomatous' OR 'liver carcinoma' OR 'liver carcinoma rupture' OR 'Liver Cell Carcinoma' OR 'Liver Cell Carcinoma, Adult' OR 'Liver Cell Carcinomas' OR 'malignant hepatoma' OR 'primary liver carcinoma'):ti,ab,kw 11622<br>2. MeSH descriptor: [Carcinoma, Hepatocellular] explode all trees 2014<br>3. ('Blockade, PD 1 PD L1' OR 'Checkpoint Blockade, Immune' OR 'Checkpoint Blockers, Immune' OR 'Checkpoint Inhibition, Immune' OR 'Checkpoint Inhibitor, Immune' OR 'Checkpoint Inhibitors, Immune' OR 'CTLA 4 Inhibitor*' OR 'Cytotoxic T Lymphocyte Associated Protein 4 Inhibitor' OR 'Cytotoxic T Lymphocyte Associated Protein 4 Inhibitors' OR

		'Immune Checkpoint Blockade' OR 'immune checkpoint blocker*' OR 'Immune Checkpoint Inhibition' OR 'Immune Checkpoint Inhibitor' OR 'Immune Checkpoint Inhibitors' OR 'Inhibitor, PD 1' OR 'PD 1 Inhibitor*' OR 'PD 1 PD L1 Blockade' OR 'PD L1 Inhibitor*' OR 'Programmed Cell Death Protein 1 Inhibitor*' OR 'Programmed Death Ligand 1 Inhibitors'):ti,ab,kw 7205<br>4. MeSH descriptor: [Immune Checkpoint Inhibitors] explode all trees 81<br>5. ('Nivolumab' OR 'Atezolizumab' OR 'Ipilimumab' OR 'Pembrolizumab' OR 'Camrelizumab' OR 'Cemiplimab' OR 'Durvalumab' OR 'Sintilimab'):ti,ab,kw 6935<br>6. ('Tyrosine kinase inhibitors' OR 'TKI' OR 'Multikinase inhibitor' OR 'Sorafenib' OR 'Lenvatinib' OR 'Regorafenib' OR 'Cabozantinib' OR 'Gefitinib' OR 'Brivanib' OR 'Dovitinib' OR 'Erlotinib' OR 'Everolimus' OR 'Linifanib' OR 'Nintedanib' OR 'Orantinib' OR 'Slunitinib' OR 'Vandetanib' OR 'Bevacizumab'):ti,ab,kw 18569<br>7. ('randomized' OR 'RCT'):ti,ab,kw 1025072<br>8. (‘Advanced’):ti,ab,kw 62384<br>9. (#1 OR #2) AND ((#3 OR #4 OR #5) OR (#6)) AND (#7) AND (#8) (940)

Supplementary Table 1. Search Strategy.

Study	Arm	Trial Size	Median age (years)	Western region (%)	ECOG 0 (%)	Child-Pugh A (%)	BCLC C (%)
SHARP	Sorafenib<br>Placebo	299<br>303	65<br>66	100%<br>100%	54%<br>54%	95%<br>98%	82%<br>83%
Asia Pacific	Sorafenib<br>Placebo	150<br>76	51<br>52	n.a.<br>n.a.	25.3%27.6%	97.3%<br>97.4%	95.3%<br>96.1%
REFLECT	Lenvatinib<br>Sorafenib	478<br>476	63<br>62	33%<br>33%	64%<br>63%	99%<br>99%	78%<br>81%
Qin 2021	Donafenib<br>Sorafenib	328<br>331	53<br>53	n.a.<br>n.a.	39%<br>33%	99%<br>96%	87%<br>88%
BRISK-FL	Brivanib<br>Sorafenib	577<br>578	61<br>60	40%<br>35%	64%<br>61%	92%<br>92%	77%<br>78%
SUN1170	Sunitinib<br>Sorafenib	530<br>544	59<br>59	24.1%<br>24.6%	52.5%<br>52.9%	99.8%<br>99.4%	87.2%<br>83.5%
Cainap 2015	Linifanib<br>Sorafenib	514<br>521	59<br>60	34%<br>32.8%	62.8%<br>66.2%	93.2%<br>95%	84.2%<br>80.4%
CheckMate459	Nivolumab<br>Sorafenib	371<br>372	65<br>65	54%<br>53%	73%<br>70%	98%<br>96%	82%<br>78%
RATIONALE-301	Tislelizumab<br>Sorafenib	342<br>332	62<br>60	89%<br>83%	53.5%<br>54.5%	100%<br>100%	79.5%<br>75.9%
IMbrave150	Atezolizumab+ bevacizumab	336	64	60%	62%	100%	82%

	Sorafenib	165	66	59%	62%	100%	81%
ORIENT-32	Sintilimab+ IBI305	380	53	n.a.	48%	96%	85%
	Sorafenib	191	54	n.a.	48%	95%	86%
COSMIC-312	Cabozantinib+ atezolizumab	432	64	72%	64%	100%	68%
	Sorafenib	217	64	71%	66%	100%	67%
SHR-1210-III-310	Camrelizumab + apatinib	272	58	17.3%	44.1%	100%	86%
	Sorafenib	271	56	17.3%	42.8%	100%	85.2%
LEAP-002	Lenvatinib+ pembrolizumab	395	66	69.4%	67.7%	99.5%	78.5%
	Lenvatinib	399	66	69.2%	68.4%	99.5%	75.7%
HIMALAYA	tremelimumab+ durvalumab	393	65	60%	62%	100%	80%
	Sorafenib	389	64	60%	62%	100%	83%

Supplementary Table 2. Baseline characteristics for patients included in the first-line trials. n.a., not available.

Study	Arm	Trial Size	Median age (years)	Western region (%)	ECOG 0 (%)	Child-Pugh A (%)	BCLC C (%)
AHELP	Apatinib<br>Placebo	261<br>132	51<br>50	n.a.<br>n.a.	25%<br>25%	95%<br>93%	89%<br>92%
BRISK-PS	Brivanib<br>Placebo	263<br>132	64<br>62	52%<br>55%	57%<br>61%	92%<br>91%	87%<br>85%
CELESTIAL	Cabozantinib<br>Placebo	470<br>237	64<br>64	60%<br>60%	52%<br>55%	98%<br>99%	n.a.<br>n.a.
KEYNOTE-240	Pembrolizumab<br>Placebo	278<br>135	67<br>65	61.5%<br>62.9%	58.3%<br>52.6%	99.6%<br>98.5%	79.9%<br>78.5%
KEYNOTE-394	Pembrolizumab<br>Placebo	300<br>153	54<br>54	15%<br>13.7%	n.a.<br>n.a.	100%<br>100%	92.3%<br>95.4%
REACH	Ramucirumab<br>Placebo	283<br>282	64<br>62	54%<br>52%	56%<br>54%	98%<br>98%	88%<br>88%
REACH-2	Ramucirumab<br>Placebo	197<br>95	64<br>64	48%<br>53%	57%<br>58%	100%<br>100%	83%<br>79%
RESORCE	Regorafenib<br>Placebo	379<br>194	64<br>62	62%<br>62%	65%<br>67%	98%<br>97%	86%<br>89%

Supplementary Table 3. Baseline characteristics for patients included in the second-line trials. n.a., not available.

Treatment	p-score
Anti-PD-1+Bev	0.95013173
Anti-PD-L1+Bev	0.93597212
Anti-PD-1+TKI	0.83310865
Anti-PD-L1+anti-CTLA-4	0.70125481
Donafenib	0.60768942
Nivolumab	0.57142788
Tislelizumab	0.57054615
Lenvatinib	0.51123846
Anti-PD-L1+TKI	0.47794327
Sorafenib	0.29693846
Linifanib	0.24198750
Brivanib	0.22030288
Sunitinib	0.06784327
Placebo	0.01361538

Supplementary Table 4. P-scores reporting the probability for each treatment of being the best in reducing the risk of death in first-line therapy. Anti-PD-1+Bev: programmed cell death 1 inhibitor combined with bevacizumab, anti-PD-L1+Bev: programmed death ligand 1 inhibitor combined with bevacizumab, anti-PD-1+TKI: programmed cell death 1 inhibitor combined with tyrosine kinase inhibitor, anti-PD-L1+TKI: programmed death ligand 1 inhibitor combined with tyrosine kinase inhibitor, anti-PD-L1+anti-CTLA-4: programmed death ligand 1 inhibitor combined with cytotoxic T cell lymphocyte antigen 4 inhibitor.

Treatment	p-score
Anti-PD-1+TKI	0.9285587
Anti-PD-1+Bev	0.8896702
Anti-PD-L1+Bev	0.8409019
Anti-PD-L1+TKI	0.7781529
Lenvatinib	0.7599058
Linifanib	0.6238298
Anti-PD-L1+anti-CTLA-4	0.4580058
Donafenib	0.4416731
Nivolumab	0.4102413
Sorafenib	0.2926067
Brivanib	0.2796500
Tislelizumab	0.1692692
Sunitinib	0.1274846
Placebo	0.0000500

Supplementary Table 5. P-scores reporting the probability for each treatment of being the best in reducing the risk of PFS events in first-line therapy. Anti-PD-1+Bev: programmed cell death 1 inhibitor combined with bevacizumab, anti-PD-L1+Bev: programmed death ligand 1 inhibitor combined with bevacizumab, anti-PD-1+TKI: programmed cell death 1 inhibitor combined with tyrosine kinase inhibitor, anti-PD-L1+TKI: programmed death ligand 1 inhibitor combined with tyrosine kinase inhibitor, anti-PD-L1+anti-CTLA-4: programmed death ligand 1 inhibitor combined with cytotoxic T cell lymphocyte antigen 4 inhibitor.

Treatment	p-score
Anti-PD-1+Bev	0.85767500
Anti-PD-1+TKI	0.84812788
Anti-PD-L1+anti-CTLA-4	0.78123173
Anti-PD-L1+TKI	0.65145673
Lenvatinib	0.64895769
Tislelizumab	0.60158558
Anti-PD-L1+Bev	0.57985769
Nivolumab	0.52340000
Donafenib	0.38673654
Linifanib	0.38350769
Brivanib	0.30264038
Sunitinib	0.22422404
Sorafenib	0.16334423
Placebo	0.04725481

Supplementary Table 6. P-scores reporting the probability for each treatment of being the best in improving ORR events in first-line therapy. Anti-PD-1+Bev: programmed cell death 1 inhibitor combined with bevacizumab, anti-PD-L1+Bev: programmed death ligand 1 inhibitor combined with bevacizumab, anti-PD-1+TKI: programmed cell death 1 inhibitor combined with tyrosine kinase inhibitor, anti-PD-L1+TKI: programmed death ligand 1 inhibitor combined with tyrosine kinase inhibitor, anti-PD-L1+anti-CTLA-4: programmed death ligand 1 inhibitor combined with cytotoxic T cell lymphocyte antigen 4 inhibitor.

Treatment	p-score
Nivolumab	0.9017552
Tislelizumab	0.7844344
Anti-PD-L1+anti-CTLA-4	0.7089542
Donafenib	0.7063437
Sorafenib	0.5611792
Anti-PD-L1+Bev	0.5437167
Brivanib	0.5133719
Anti-PD-1+Bev	0.4375260
Sunitinib	0.3619719
Lenvatinib	0.3155677
Linifanib	0.2886250
Anti-PD-L1+TKI	0.2305365
Anti-PD-1+TKI	0.1460177

Supplementary Table 7. P-scores reporting the probability for each treatment of being the best in reducing the risk of ⩾3AE events in first-line therapy. Anti-PD-1+Bev: programmed cell death 1 inhibitor combined with bevacizumab, anti-PD-L1+Bev: programmed death ligand 1 inhibitor combined with bevacizumab, anti-PD-1+TKI: programmed cell death 1 inhibitor combined with tyrosine kinase inhibitor, anti-PD-L1+TKI: programmed death ligand 1 inhibitor combined with tyrosine kinase inhibitor, anti-PD-L1+anti-CTLA-4: programmed death ligand 1 inhibitor combined with cytotoxic T cell lymphocyte antigen 4 inhibitor.

Treatment	p-score
Regorafenib	0.94646667
Cabozantinib	0.64001667
Pembrolizumab	0.56357292
Apatinib	0.56075417
Ramucirumab	0.45989375
Brivanib	0.29197292
Placebo	0.03732292

Supplementary Table 8. P-scores reporting the probability for each treatment of being the best in reducing the risk of death in second-line therapy.

Treatment	p-score
Cabozantinib	0.87355833
Regorafenib	0.79886875
Apatinib	0.74588125
Brivanib	0.47952917
Ramucirumab	0.42198750
Pembrolizumab	0.18015625
Placebo	0.00001875

Supplementary Table 9. P-scores reporting the probability for each treatment of being the best in reducing the risk of PFS events in second-line therapy.

Treatment	p-score
Cabozantinib	0.7042354
Ramucirumab	0.6349833
Apatinib	0.6317333
Brivanib	0.6081813
Pembrolizumab	0.5803042
Regorafenib	0.2974500
Placebo	0.0431125

Supplementary Table 10. P-scores reporting the probability for each treatment of being the best in improving ORR events in second-line therapy.

Treatment	p-score
Placebo	0.9014175
Pembrolizumab	0.7047450
Cabozantinib	0.5056200
Regorafenib	0.4117575
Brivanib	0.3251000
Apatinib	0.1513600

Supplementary Table 11. P-scores reporting the probability for each treatment of being the best in reducing the risk of ⩾3AE events in second-line therapy.

Study	Arm	mOS (months) (95%CI)	HR (95%CI) *p* value	mPFS (months) (95%CI)	HR (95%CI) *p* value	ORR (%) (95%CI)
**SHARP**	**Sorafenib**	**10.7 (9.4-13.3)**	**0.69 (0.6-0.9)**	**4.1 (3.5-4.8)**	**1.1 (0.9-1.3)**	**2**
	**Placebo**	**7.9 (6.8-9.1)**	**<0.001**	**4.9 (4.2-6.3)**	**0.77**	**1**
**Asia Pacific**	**Sorafenib**	**6.5 (5.6-7.6)**	**0.68 (0.5-0.9)**	**2.8 (2.6-3.6)**	**0.57 (0.4-0.8)**	**3.3**
	**Placebo**	**4.2 (3.8-5.5)**	**0.014**	**1.4 (1.3-1.6)**	**0.0005**	**1.3**
**REFLECT**	**Lenvatinib**	**13.6 (12.1-14.9)**	**0.92 (0.8-1.1)**	**7.3 (5.6-7.5)**	**0.65 (0.6-0.8)**	**18.8 (15.3-22.3)**
	**Sorafenib**	**12.3 (10.4-13.9)**			**<0.0001**	**6.5 (4.3-5.14)**
**Qin 2021**	**Donafenib**	**12.0 (10.3-13.1)**	**0.84 (0.7-0.9)**	**3.7 (3.0-3.7)**	**0.91 (0.8-1.1)**	**4.6**
	**Sorafenib**	**10.1 (9.2-11.9)**	**0.31**	**3.6 (2.4-3.7)**	**0.057**	**2.7**
**BRISK-FL**	**Brivanib**	**9.9 (8.5-11.5)**	**1.07 (0.9-1.2)**	**4.1 (3.1-4.2)**	**1.01 (0.9-1.2)**	**12 (9-15)**
	**Sorafenib**	**9.5 (8.3-10.6)**	**0.31**	**4.2 (4.1-4.3)**	**0.85**	**9 (7-11)**
**SUN1170**	**Sunitinib**	**7.9 (7.4-9.2)**	**1.30 (1.1-1.5)**	**3.6 (2.8-4.1)**	**1.13 (0.9-1.3)**	**6.2**
	**Sorafenib**	**10.2 (8.9-11.4)**	**0.99**	**3.0 (2.8-4.0)**	**0.88**	**5.9**
**Cainap 2015**	**Linifanib**	**9.1 (8.1-10.2)**	**1.05 (0.9-1.2)**	**5.4 (4.2-5.6)**	**0.76 (0.6-0.9)**	**10.1**
	**Sorafenib**	**9.8 (8.3-11)**		**4.0 (2.8-4.2)**	**0.001**	**6.1**
**CheckMate459**	**Nivolumab**	**16.4 (13.9-18.4)**	**0.85 (0.7-1.0) 0.075**	**3.7 (3.1-3.9)**	**0.93 (0.8-1.1)**	**15 (12-19)**
	**Sorafenib**	**14.7 (11.9-17.2)**		**3.8 (3.7-4.5)**		**7 (5-10)**
**RATIONALE-301**	**Tislelizumab**	**15.9**	**0.85 (0.7-1.0) 0.0398**	**2.2**	**1.1 (0.9-1.3)**	**14.3 (10.8-18.5)**
	**Sorafenib**	**14.1**		**3.6**		**5.4 (3.2-8.4)**
**IMbrave150**	**Atezolizumab+bevacizumab**	**19.2 (17-23.7)**	**0.66 (0.5-0.9)**	**6.9 (4.7-8.6)**	**0.65 (0.53-0.81)**	**30.0 (25.0-35.0)**
	**Sorafenib**	**13.4 (11.4-16.9)**	**<0.001**	**4.3 (4.0-5.6)**	**<0.001**	**11.0 (7.0-17.0)**

**ORIENT-32**	**Sintilimab+IBI305**	**NR**	**0.57 (0.4-0.8)**	**4.6 (4.1-5.7)**	**0.56 (0.5-0.7)**	**21 (17-25)**
	**Sorafenib**	**10.4 (8.5-NR)**	**<0.0001**	**2.8 (2.7-3.2)**	**<0.0001**	**4 (2–8)**
**COSMIC-312**	**Cabozantinib+atezolizumab**	**15.4 (13.7-17.7)**	**0.90 (0.7-1.2)**	**6.8 (5.6-8.3)**	**0.63 (0.4-0.9)**	**11.2 (8.1-14)**
	**Sorafenib**	**15.5 (12.1-NR)**	**0.438**	**4.2 (2.8-7.0)**	**0.0012**	**3.7 (1.6-7.1)**
**SHR-1210-III-310**	**Camrelizumab+ apatinib**	**22.1 (19.1-27.2)**	**0.62 (0.5-0.8)**	**5.6 (5.5-6.3)**	**0.52 (0.4-0.7)**	**25.4 (20.3-31)**
	**Sorafenib**	**15.2 (13.0-18.5)**	**<0.0001**	**3.7 (2.8-3.7)**	**<0.0001**	**5.9 (3.4-9.4)**
**LEAP-002**	**Lenvatinib+pembrolizumab**	**21.2 (19.0-23.6)**	**0.84 (0.7-0.9)**	**8.2 (6.4-8.4)**	**0.87 (0.7-1.0)**	**26.1 (21.8-30.7)**
	**Lenvatinib**	**19.0 (17.2-21.7)**	**0.0227**	**8.0 (6.3-8.2)**	**0.0466**	**17.5 (13.9-21.6)**
**HIMALAYA**	**Tremelimumab+durvalumab**	**16.4 (14.2-19.6)**	**0.78 (0.7-0.9)**	**3.8 (3.7-5.3)**	**0.90 (0.8-1.1)**	**20.1**
	**Sorafenib**	**13.8 (12.3-16.1)**	**0.0035**	**4.2 (3.8-5.5)**		**5.1**

Supplementary Table 12. Descriptive report of efficacy outcomes in the first-line trials. mOS, median overall survival; mPFS, median progression-free survival; NR, not reached; ORR, objective response rate.

Study	Arm	mOS (months) (95%CI)	HR (95%CI) *p* value	mPFS (months) (95%CI)	HR (95%CI) *p* value	ORR (%) (95%CI)
AHELP	Apatinib	8.7 (7.5-9.8)	0.785 (0.617-0.998)	4.5 (3.9-4.7)	0.471 (0.369-0.601)	11
	Placebo	6.8 (5.7-9.1)	0.048	1.9 (1.9-2.0)	<0.0001	2
BRISK-PS	Brivanib	9.4	0.89 (0.69-1.15)	n.a.	n.a.	9.9
	Placebo	8.2	0.3307	n.a.		1.5
CELESTIAL	Cabozantinib	10.2 (9.1-12)	0.76 (0.63-0.92)	5.2 (4.0-5.5)	0.44 (0.36-0.52)	3.8
	Placebo	8 (6.8-9.4)	0.005	1.9 (1.9-2.0)	<0.001	0.4
KEYNOTE-240	Pembrolizumab	13.9 (11.6-16)	0.78 (0.61-1)	3 (2.8-4.1)	0.72 (0.57-0.9)	18.3 (14-23.4)
	Placebo	10.6 (8.3-13.5)	0.0238	2.8 (1.6-3.0)	0.0022	4.4 (1.6-9.4)
KEYNOTE-394	Pembrolizumab	14.6 (12.6-18)	0.79 (0.63-0.99)	2.6 (1.5-2.8)	0.74 (0.6-0.92)	13.7
	Placebo	13 (10.5-15.1)	0.018	2.3 (1.4-2.8)	0.0032	1.3
REACH	Ramucirumab	9.2 (8.0-10.6)	0.87 (0.72-1.05)	2.8 (2.7-3.9)	0.63 (0.52-0.75)	7.1
	Placebo	7.6 (6.0-9.3)	0.14	2.1 (1.6-2.7)	<0.001	0.7
REACH-2	Ramucirumab	8.5 (7.0-10.6)	0.71 (0.53-0.95)	2.8 (2.8-4.1)	0.452 (0.339-0.603)	5
	Placebo	7.3 (5.4-9.1)	0.0199	1.6 (1.5-2.7)	<0.0001	1
RESORCE	Regorafenib	10.6 (9.1-12.1)	0.63 (0.5-0.79)	3.1 (2.8-4.2)	0.46 (0.37-0.56)	11
	Placebo	7.8 (6.3-8.8)	<0.0001	1.5 (1.4-1.6)	<0.0001	4

Supplementary Table 13. Descriptive report of efficacy outcomes in the second-line trials. mOS, median overall survival; mPFS, median progression-free survival; n.a., not available; NR, not reached; ORR, objective response rate.

Study	Arm	Any grade AE, n (%)	Grade ⩾3 AEs, n (%)
SHARP	Sorafenib<br>Placebo	98%<br>96%	45%<br>32%
Asia Pacific	Sorafenib<br>Placebo	146 (98%)<br>71 (94.7%)	71 (serious) (47.7%)<br>34 (serious) (45.3%)
REFLECT	Lenvatinib<br>Sorafenib	470 (99%)<br>472 (99%)	357 (75%)<br>316 (67%)
Qin 2021	Donafenib<br>Sorafenib	332 (100%)<br>329 (99%)	191 (57%)<br>224 (67%)
BRISK-FL	Brivanib<br>Sorafenib	564 (98%)<br>569 (99%)	384 (68%)<br>371 (65.2%)
SUN1170	Sunitinib<br>Sorafenib	n.a.<br>n.a.	449 (85.4%)<br>404 (74.5%)
Cainap 2015	Linifanib<br>Sorafenib	508 (99.6%)<br>511 (98.5%)	435 (85.3%)<br>389 (75%)
CheckMate459	Nivolumab<br>Sorafenib	257 (70%)<br>338 (93.1%)	82 (22.3%)<br>180 (49.5%)
RATIONALE-301	Tislelizumab<br>Sorafenib	325 (96.2%)<br>324 (100%)	163 (48.2%)<br>212 (65.4%)
IMbrave150	Atezolizumab+bevacizumab<br>Sorafenib	322 (98%)<br>154 (99%)	230 (69.9%)<br>98 (62.8%)
ORIENT-32	Sintilimab+IBI305	376 (99%)	207 (54%)

	**Sorafenib**	**181 (98%)**	**87 (47%)**
**COSMIC-312**	**Cabozantinib+atezolizumab**<br><br>**Sorafenib**	**93%**<br><br>**90%**	**63.5%**<br><br>**41%**
**SHR-1210-III-310**	**Camrelizumab+ apatinib**<br><br>**Sorafenib**	**265 (97.4%)**<br><br>**249 (92.6%)**	**220 (80.9%)**<br><br>**142 (52.8%)**
**LEAP-002**	**Lenvatinib+pembrolizumab**<br><br>**Lenvatinib**	**381 (96.5%)**<br><br>**378 (95.7%)**	**247 (62.5%)**<br><br>**227 (57.5%)**
**HIMALAYA**	**tremelimumab+durvalumab**<br><br>**Sorafenib**	**378 (97.4%)**<br><br>**357 (95.5%)**	**196 (50.5%)**<br><br>**196 (52.4%)**

Supplementary Table 14. Descriptive incidence of AEs in the first-line trials. AE, adverse event; n.a., not available.

Study	Arm	Any grade AE, n (%)	Grade ⩾3 AEs, n (%)
AHELP	Apatinib	97	77
	Placebo	71	25
BRISK-PS	Brivanib	94	68
	Placebo	62	24
CELESTIAL	Cabozantinib	99	68
	Placebo	92	36
KEYNOTE-240	Pembrolizumab	96.4	52.7
	Placebo	90.3	46.3
KEYNOTE-394	Pembrolizumab	66.9	14.4
	Placebo	49.7	5.9
REACH	Ramucirumab	n.a.	n.a.
	Placebo	n.a.	n.a.
REACH-2	Ramucirumab	n.a.	n.a.
	Placebo	n.a.	n.a.
RESORCE	Regorafenib	93	50
	Placebo	52	17

Supplementary Table 15. Descriptive incidence of AEs in the second-line trials. AE, adverse event; n.a., not available.

Supplementary Figures

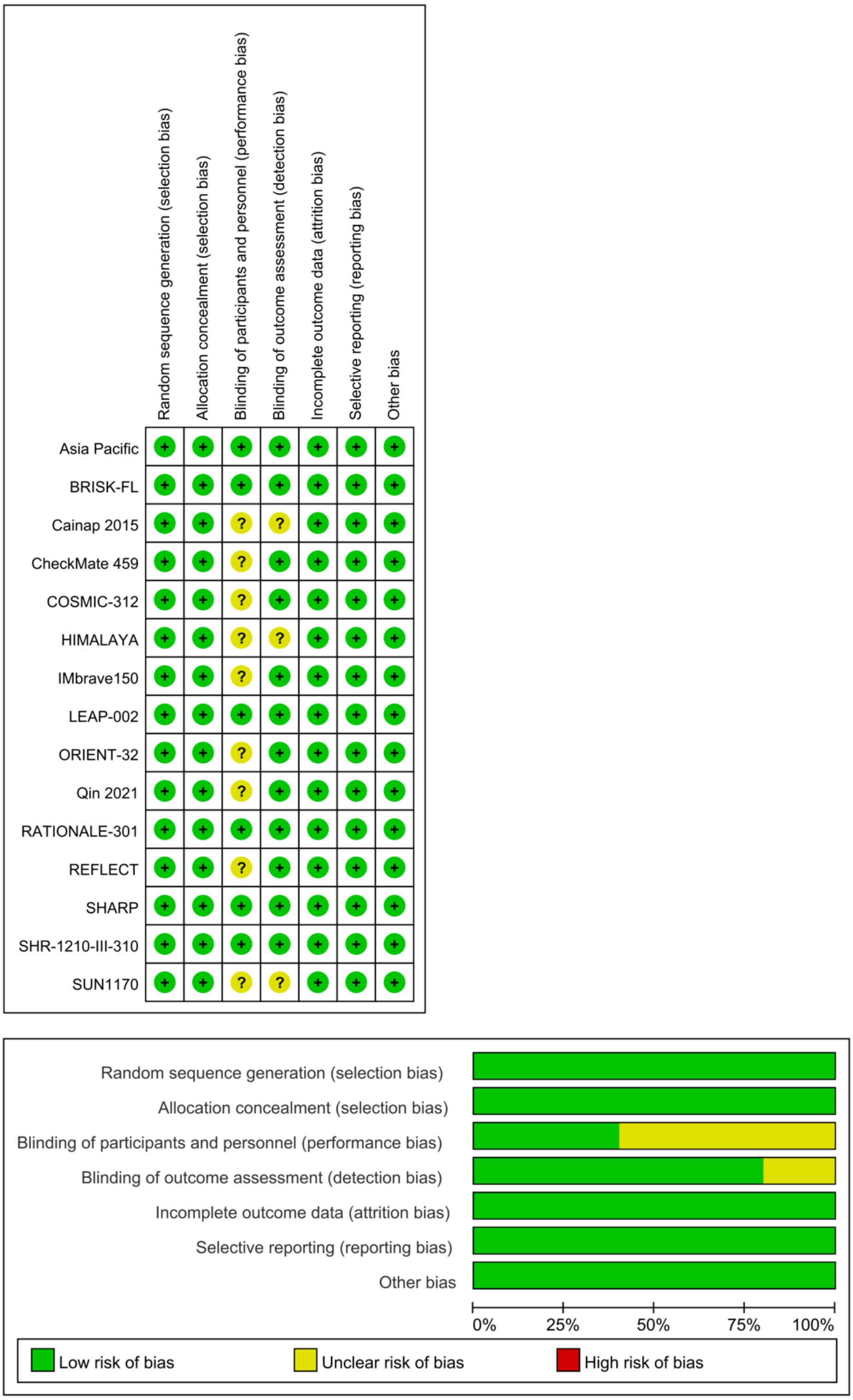

Supplementary Figure 1. Risk of bias graph for first-line studies according to the Cochrane risk of bias assessment tool.

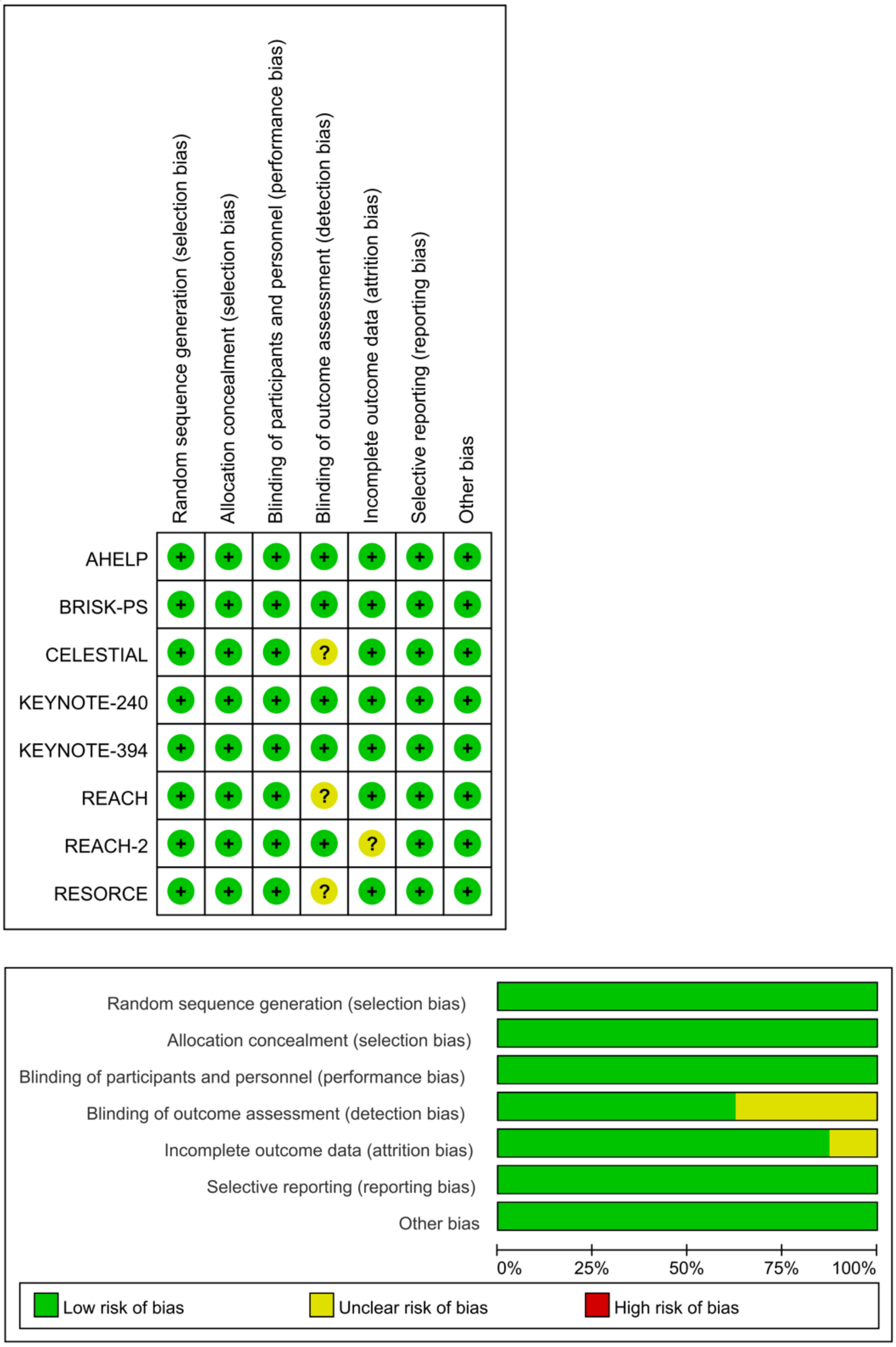

Supplementary Figure 2. Risk of bias graph for second-line studies according to the Cochrane risk of bias assessment tool.
